# The complete chloroplast genome of *Dendrobium longicornu* (Orchidaceae)

**DOI:** 10.1080/23802359.2019.1666049

**Published:** 2019-10-25

**Authors:** Xin-Yi Wu, Ting-Zhang Li, Gui-Zhen Chen, Qing Xu, Yun-Yun Pan, Li Jun Chen

**Affiliations:** a Key Laboratory of National Forestry and Grassland Administration for Orchid Conservation and Utilization, Shenzhen, China;; b Shenzhen Key Laboratory for Orchid Conservation and Utilization, The National Orchid Conservation Centre of China, The Orchid Conservation and Research Centre of Shenzhen, Shenzhen, China;; c GMU-GIBH Joint School of Life Sciences, Guangzhou Medical University, Guangzhou, China

**Keywords:** *Dendrobium longicornu*, chloroplast genome, phylogenetic, Illumina sequencing

## Abstract

*Dendrobium longicornu* Lindl is an epiphytic orchid with significant ornamental values. Here, we report the first complete chloroplast genome of *D. longicornu*. The complete chloroplast (cp) genome sequence of *D. longicornu* is 160,024 bp in length and consisted of two inverted repeats (IRs, 25,403 bp), which were separated by a large single copy region (LSC, 88,075 bp) and a small single copy region (SSC, 21,143 bp). The cp genome encoded 142 genes, of which 110 were unique genes (80 protein-coding genes, 26 tRNAs and 4 rRNAs). Phylogenetic analysis showed that *D. longicornu* clustered together with *D. ellipsophy*.

*Dendrobium* Swartz (1799), the second largest genera in Ochidaceae, over 1450 species, are distributed in tropical and subtropical regions of Asia and Oceania(Cribb and Govaerts 2005; Pridgeon et al. 2014). Most of the species in this genus are epiphytes with high ornamental, medicinal and commercial values and are favored by botanists and plant enthusiasts. However, the phylogenetic relationships in *Dendrobium* are unclear.

*Dendrobium longicornu* Lindl (1830) is an epiphytic orchid, which grows on tree trunks in mountain forests and its native range is Himalaya to China. For the first time, we sequenced the complete plastome of *D*. *longicornu*, and assessed phylogenetic position within *Dendrobium*.

Leaf samples of *D. longicornu* were obtained from the Orchid Conservation and Research Centre of Shenzhen and specimens were deposited in the National Orchid Conservation Center herbarium (NOCC; specimen code Z.J.Liu 6503). Total genomic DNA was extracted from fresh leaves using the modified CTAB procedure method (Doyle and Doyle 1987) and sequenced by using Illumina Hiseq 4000 platform (San Diego, CA). Genome sequences were screened out and assembled with MITObim v1.8 (Hahn et al. 2013). After assembled, the obtained scaffolds and contigs were annotated with CpGAVAS (Liu et al. 2012) then adjusted by Geneious version 11.1.15 (Kearse et al. 2012) and submitted to GenBank with accession number MN227146.

The cp genome sequence of *D. longicornu* is 160,024 bp length and presented a typical quadripartite structure including one large single-copy region (LSC, 88,075 bp), one small single-copy region (SSC, 21,143 bp), and two inverted repeat regions (IRs, 25,403 bp). The cp genome encoded 142 genes, of which 110 were unique genes (80 protein-coding genes, 26 tRNAs and 4 rRNAs).

To further investigate its phylogenetic position, we used 39 accessions of *Dendrobium* for molecular analysis. RAxML-HPC2 on XSEDE 8.2.10 (Stamatakis et al. 2008) was used to construct a maximum likelihood tree, and two species of *Pleione* was used as outgroup. The branch support was computed with 1000 bootstrap replicates and settings as described by Stamatakis et al. (2008). Phylogenetic analysis showed that *D. longicornu* is sister with *D. ellipsophy* (Figure 1). The determination of the complete plastid genome sequences provided new molecular data to illuminate the *Dendrobium* evolution.

**Figure 1.**
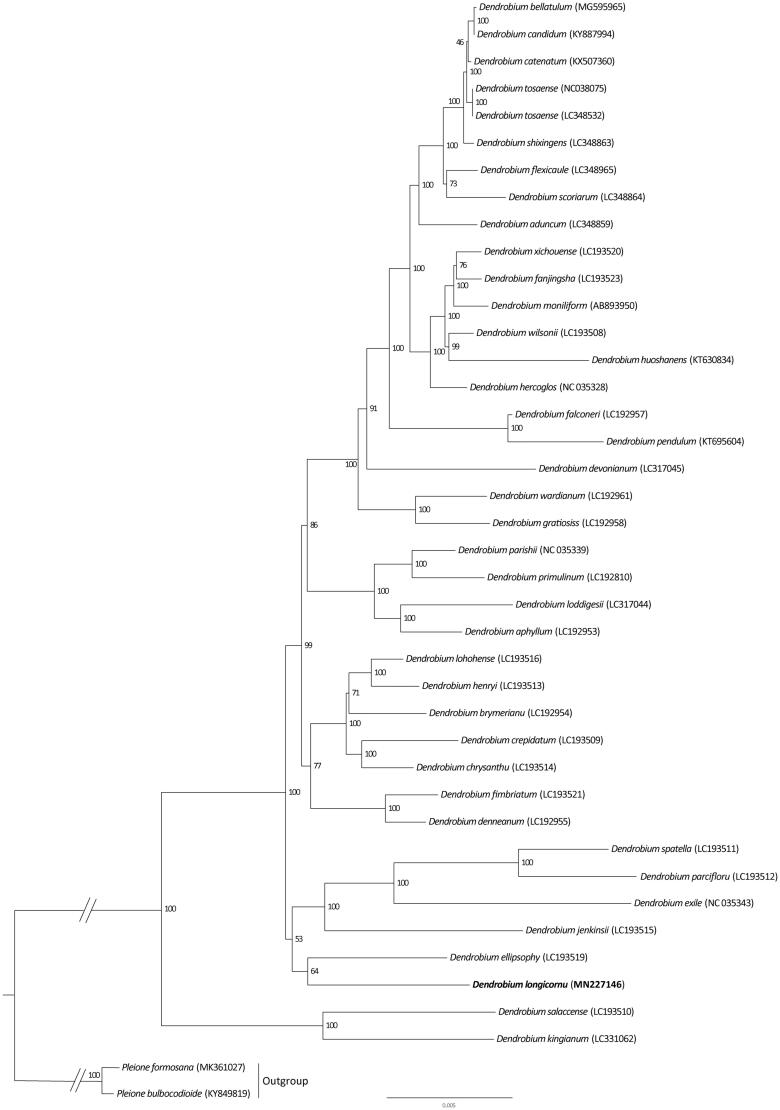
Phylogenetic position of *Dendrobium longicornu* inferred by maximum likelihood (ML) of complete cp genome. The bootstrap values are shown next to the nodes.
